# Antioxidant Capacity Assessment of Plant Extracts for Green Synthesis of Nanoparticles

**DOI:** 10.3390/nano11071679

**Published:** 2021-06-25

**Authors:** María Martínez-Cabanas, Marta López-García, Pilar Rodríguez-Barro, Teresa Vilariño, Pablo Lodeiro, Roberto Herrero, José L. Barriada, Manuel E. Sastre de Vicente

**Affiliations:** 1 Chemical Oceanography, Marine Biogeochemistry, GEOMAR Helmholtz Centre for Ocean Research Kiel, 24148 Kiel, Germany; mmartinez@geomar.de; 2 Amphos 21 Consulting S.L. Carrer Veneçuela 103, 2-1., 08019 Barcelona, Spain; marta.lopez@amphos21.com; 3 Departamento de Química and Centro de Investigaciones Científicas Avanzadas (CICA), Universidade da Coruña, rúa da fraga 10, 15071 A Coruña, Spain; pilar.rbarro@udc.es (P.R.-B.); teresa.vilarino@udc.es (T.V.); r.herrero@udc.es (R.H.); manuel.sastre@udc.es (M.E.S.d.V.); 4 Department of Chemistry, University of Lleida—AGROTECNIO-CERCA Center, Rovira Roure 191, 25198 Lleida, Spain; pablo.lodeiro@udl.cat

**Keywords:** plant extracts, agricultural waste utilization, antioxidant capacity, metallic nanoparticles

## Abstract

In this work, water extracts from different bio-based products of plant origin were studied to evaluate their antioxidant capacity and their potential to form metal nanoparticles from aqueous solutions. Two traditional tests, the Folin–Ciocalteu assay and the DPPH radical scavenging capacity method were compared with a more recent one, SNPAC, based on the formation of silver nanoparticles. The silver nanoparticle antioxidant capacity method (SNPAC) was optimized for its application in the characterization of the extracts selected in this work; kinetic studies and extract concentration were also evaluated. The extracts were obtained from leaves of oak, eucalyptus, green tea, white and common thyme, white cedar, mint, rosemary, bay, lemon, and the seaweed *Sargassum muticum*. The results demonstrate that any of these three methods can be used as a quick test to identify an extract to be employed for nanoparticle formation. Additionally, we studied the synthesis of Cu, Fe, Pb, Ni, and Ag nanoparticles using eucalyptus extracts demonstrating the efficiency of this plant extract to form metallic nanoparticles from aqueous metal salt solutions. Metal nanoparticles were characterized by transmission electron microscopy and dynamic light scattering techniques.

## 1. Introduction

The U.S. Environmental Protection Agency define Nanotechnology as the research and control of matter with a scale between 1 and 100 nm, and the creation and use of structures with novel properties coming from their small size [1]. The small size of the nanoparticles (NPs) results in large surface areas, high reactivity, and the tunable nature of their properties. Those facts favor the application of manufactured NPs in many areas of knowledge such as medicine, cosmetics, food, paints, electronics, and environmental remediation [2,3,4].

One of the most common methods of NP synthesis, in particular for metallic NPs, is the reduction of metal salts in solution. Synthesis of monodisperse NPs requires control over the parameters affecting the growth rate of the NPs, e.g., concentration of metal salts, viscosity of solvent, and strength of reducing agents. In addition, a stabilizing agent is usually needed to avoid the aggregation of the synthesized NPs. The capping agents, e.g., organic molecules, polymers, or biological molecules, provide electrostatic stabilization by developing surface charge on the nanoparticle surface, and/or steric stabilization by anchoring long-chain hydrocarbons on the nanoparticle surface [5,6,7]. Besides reduction reactions, other techniques such as UV irradiation, lithography, or photochemical reduction have also been used with successful results. Despite the good results, these synthesis techniques remain expensive and most of the reactants used are toxic to the environment and the living systems.

In direct contrast to traditional techniques of NPs production, green synthesis methods are based on the use of biocompatible reagents that reduce the toxicity of the manufactured NPs and the environmental impact of the by-products [8]. Environmentally friendly techniques produce NPs using microorganisms [9,10], plant extracts [11,12,13,14], or marine algae [15,16,17,18,19] as reducing agents. The microorganism-mediated synthesis is slower, more expensive, and more complicated than the synthesis using plant extracts. Besides, aseptic conditions are needed during the process, and some of the waste products are dangerous to the environment. The synthesis of NPs using plant extracts is a simple and effective procedure, which involves the mixture of plant extracts and metal salt solutions. Generally, the reaction takes place at room temperature during a short period of time (from minutes to hours). The process is very cheap, and the environmental impact is reduced. Moreover, in some cases, it can constitute a revalorization of a waste product [20].

Plant extracts play a double role in the NPs synthesis process, while mediating the reduction of metal salts; they can also act as capping agents to stabilize the produced NPs. Bioreduction with plants is a complex process where a large variety of plant components like terpenoids, flavonoids, phenols, alkaloids, saponins, or proteins are involved. The antioxidant behavior of these metabolites is well known. Antioxidants are compounds capable of delaying or inhibiting the oxidation processes which occur under the influence of atmospheric oxygen or reactive oxygen species [21,22]. Results from FT-IR studies show that hydroxyl, carbonyl, or amine functional groups are the main components responsible for reducing the metal ions and also capping around the synthesized nanoparticles [23,24]. The composition of the extracts determines the characteristics of synthesized NPs (e.g., size, shape, and yield), as each extract contains different concentrations and combinations of reducing agents. Thus, the complex nature of the extracts hinders the understanding of the mechanistic process involved in nanoparticle formation [25,26,27]. In fact, in many cases, the same extract produces NPs with only one or two metals [20,28].

The diversity of natural antioxidants makes it challenging to quantify them individually in a complex matrix such as a plant extract [29,30]. Therefore, different assays to determine the total antioxidant capacity of natural matrices are employed. There are three types of methods for the assessment of total antioxidant capacities: (1) spectrometric techniques such as the 2,2-diphenyl-1-picrylhydrazyl (DPPH) radical scavenging capacity assay, the Ferric reducing antioxidant power method (FRAP), and the Folin–Ciocalteu (F–C) method; (2) electrochemical techniques such as cyclic voltammetry; and (3) chromatographic techniques such as HPLC. None of those methods can be considered universal or most suitable than the others. Each antioxidant assay has a different mechanism, reaction media, and experimental conditions. It is therefore strongly recommended to use a combination of different methods to determine the antioxidant capacity of natural compounds [22,31,32].

The main objective of this study was to demonstrate the potential of plant extracts as an alternative to hazardous chemicals in the synthesis of nanoparticles. We investigated the antioxidant capacity of plant extracts obtained from oak, eucalyptus, green tea, white thyme, common thyme, white cedar, mint, rosemary, bay and lemon leaves, and the seaweed *Sargassum muticum*. Results obtained with two traditional methods (Folin–Ciocalteu and DPPH scavenging assays) are compared to results obtained with an alternative technique, the silver nanoparticle antioxidant capacity method (SNPAC). The SNPAC method allows the determination of plant extracts reducing power measuring the formation of silver nanoparticles. Firstly, the extracts were characterized; then, the synthesis of metallic nanoparticles was tested using several metal salts and the extract which showed the best reducing power under our experimental conditions. The obtained NPs were characterized in terms of size distribution and shape.

## 2. Materials and Methods

Folin–Ciocalteus’s Reagent for clinical diagnosis, gallic acid 1-hydrate, 99% for synthesis, and sodium carbonate anhydrous for analysis (Panreac Química S.A., Barcelona, Spain) were used for total phenols determination of the extracts. The DPPH assay was carried out using 2,2-diphenyl-1-picrylhydrazyl, (±)-6-hydroxy-2,5,7,8-tetramethylchromane-2-carboxylic acid (Sigma-Aldrich Química, S.L, Madrid, Spain) and methanol (Panreac, pa. pro analysis). Sodium citrate dihydrate (J.T. Baker B.V., Deventer, Holland) was used as reductant in the SNPAC method. Silver nitrate, iron (III) nitrate 9-hydrate and nickel (II) nitrate 6-hydrate (Panreac, pa.), and lead (II) nitrate and copper (II) nitrate 3-hydrate (MERCK, Darmstadt, Germany, pa.) were used for the synthesis of metal nanoparticles.

Bio-based products used in this study were collected in Galicia (North-West of Spain) except common and white thyme, which were collected in Argañín de Sayago (Arribes del Duero, Zamora, Middle-West of Spain). Firstly, the materials were washed with tap water to eliminate impurities and oven-dried at 60 °C overnight, except green tea. Green tea was used directly as purchased and also exhausted (after a preparation of a tea to prove the antioxidant capacity of used tea leaves). Then, the materials were ground with an analytical mill (IKA A 10, IKA^®^ Werke GmbH & Co. KG, Staufen, Germany), sieved and stored in polyethylene flasks. Shredded bio-based materials with size between 0.5 and 1 mm were chosen to prepare the extracts.

### 2.1. Preparation of Plant Extracts

A total of 1 g of dry bio-based product was mixed with 100 mL of deionized water (18.2 MΩ·cm, MilliQ, Millipore, Molsheim, France). The extraction was carried out by reflux for 40 min. Then, supernatant solution was allowed to reach room temperature and vacuum filtration was used to separate the solid part from the extract. Eleven extracts were obtained from different plant leaves: oak (*Quercus robur*), eucalyptus (*Eucalyptus globulus*), green tea (*Camellia sinensis*), white thyme (*Thimus mastichina*), common thyme (*Thimus vulgaris*), white cedar (*Thuja occidentalis*), mint (*Mentha* sp.), rosemary (*Rosmarinus officinalis*), laurel or bay laurel (*Laurus nobilis*), and lemon tree (*Citrus limon*). An extract from the brown algae *Sargassum muticum* was also studied. Plants extracts were stored in the fridge until use. All the experiments were done at least in duplicate.

### 2.2. Total Antioxidant Capacity Determination

#### 2.2.1. Folin–Ciocalteu Method

An aliquot of each extract (0.5 mL) was diluted with 3.75 mL of deionized water. Then, 0.25 mL of Folin–Ciocalteus’s Reagent (1:1 *v*/*v*) was added and the mixture was shaken at room temperature in a vortex mixer (VELP Scientifica, Milan, Italy); 0.5 mL of 10% Na_2_CO_3_ were also added, the mixture was stirred again, and after 45 min, the absorbance was determined at 765 nm using an UV-Vis spectrophotometer (ZUZI Spectrophotometer Model 4211/20, Auxilab S.L., Navarra, Spain). The appearance of a blue color in the mixture indicated the presence of phenolic compounds. The total phenolic content in the extracts is expressed as mmol·L^−1^ of gallic acid equivalents (GAE) [33].

#### 2.2.2. DPPH Radical Scavenging Capacity

These assays were developed following the method proposed by Brand–Williams and co-workers [34]; 3 mL of a 0.06-mM DPPH solution (violet color) in methanol were mixed with 75 µL of each extract. The mixture was vigorously stirred in a vortex mixer and the absorbance was determined at 515 nm using an UV-Vis spectrophotometer (ZUZI Spectrophotometer Model 4211/20). Several kinetic assays were done to determine that the average reaction time of the tested extracts with DPPH radical was 40 min. The antioxidant capacity of each extract was shown through a change in the mixture color, from violet to pale yellow. A natural antioxidant, the (±)-6-Hydroxy-2,5,7,8-tetramethyl-chromane-2-carboxylic acid (trolox), was used to obtain a calibration curve. The antioxidant capacities are expressed as mmol·L^−1^ of trolox equivalents (TE).

#### 2.2.3. Silver Nanoparticle Antioxidant Capacity (SNPAC) Method

A volume of 150 mL of AgNO_3_ 1 mmol·L^−1^ solution was boiled for 10 min to obtain silver seeds particles [35]. Then, 15 mL of sodium citrate were added dropwise to the silver solution. The mixture was stirred vigorously and softly heated until the appearance of a pale-yellow color. Subsequently, 2 mL of the silver seeds solution were mixed with an aliquot (x mL) of each extract and (0.8 − x) mL of water. The mixtures were shaken at room temperature, and after the nanoparticle formation, the absorbance was determined at 423 nm using a UV-Vis spectrophotometer (CARY 100 BIO, Varian, Mulgrave, Australia).

### 2.3. Nanoparticle Characterization

Transmission Electron Microscopy (TEM) images were obtained to analyze the morphology of the synthesized nanoparticles. Measurements were performed using a transmission electron microscope (JEM 1010, Jeol GmbH, München, Germany).

Dynamic light scattering (DLS) analyses were done to determine the size distribution of the nanoparticles obtained from plant extracts. Measurements were performed using a Zetasizer Nano ZS (Malvern Instruments Ltd., Worcester, UK).

### 2.4. Synthesis of Metal Nanoparticles

A volume of 50 mL of a 0.1 M metal salt (AgNO_3_, Fe(NO_3_)_3_·9H_2_O, Cu(NO_3_)_2_, Ni(NO_3_)_2_·6H_2_O or Pb(NO_3_)_2_) was mixed with 50 mL of the eucalyptus extract. Metallic NP formation was followed by the color change of the metal salt solution. The synthesis was carried out at 25 °C.

## 3. Results and Discussion

### 3.1. Determination of the Total Antioxidant Capacity of the Extracts

#### 3.1.1. DPPH Radical Scavenging Capacity and Folin–Ciocalteu Methods

The DPPH radical scavenging capacity method is based on the capacity of antioxidant compounds to reduce the stable free radical 2,2-diphenyl-1-picrylhydrazyl (DPPH) to the respective hydrazine [36]. The reduction of the radical is shown by the disappearance of its violet color, which is followed through the decrease in absorbance measured at 515 nm. This method follows a radical reaction, thus the antioxidant capacity determined is likely related to the radical removal. According to the DPPH analyses, the highest antioxidant capacity was obtained from the oak, eucalyptus, green tea, and white thyme extracts, with values above 5 TE (mM trolox). In contrast, *S. muticum*, mint, and laurel extracts presented the smallest total antioxidant capacity (<1 TE).

The total polyphenol content was obtained for each extract following the method proposed by Folin and Ciocalteu [37] and improved by Singleton and Rossi in 1965 [33]. This method is based on the reduction of the Folin reagent, a molybdotungstophosphoric heteropolyanion, by phenols, but also other reducing compounds in the sample, leading to a blue product with an absorbance maximum at 765 nm. Theoretically, F–C measurements give the amount of total phenols contained in the samples. Nevertheless, the reaction which takes place is not selective [38]. We therefore hypothesize that the reduction of the F–C reagent is carried out not only by phenols, but also by other reducing agents in the sample. The total phenolic content of oak, eucalyptus, white thyme, and green tea extracts presented enhanced values of above 4 GAE (mM gallic acid), whilst the mint, laurel, and algae extracts showed the lowest phenolic concentration (<2 GAE).

**Figure 1:**
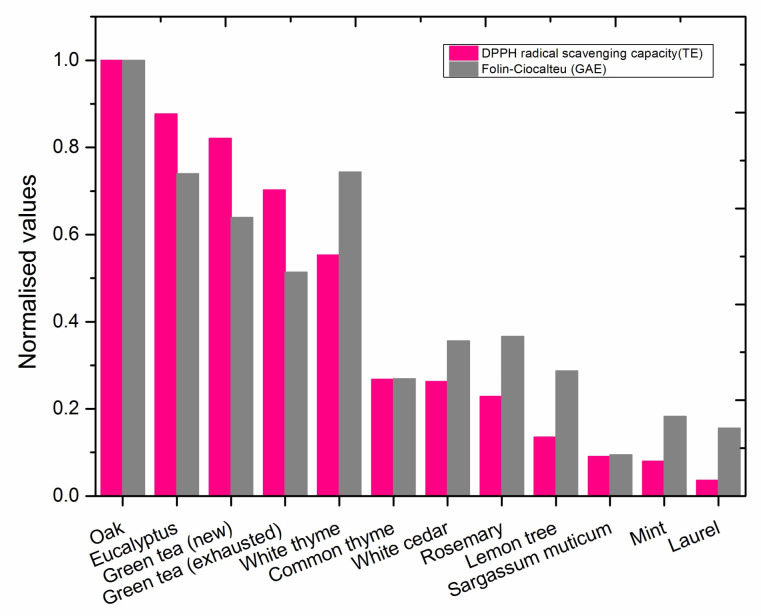
Determination of total antioxidant capacity of plant extracts with traditional methods: Folin–Ciocalteu and DPPH radical scavenging capacity. Normalized values respect the maximum value of each assay.

Normalized values obtained by F–C and DPPH assays are compared in Figure 1. Data were normalized with respect to the maximum values of the two assays, the oak extract value (F–C = 8.13 GAE; DPPH = 9.59 TE). Data obtained with both F–C and DPPH analyses show a similar trend. We grouped the extracts according to their total antioxidant capacity. The group with the highest reducing power includes oak, eucalyptus, white thyme, and green tea extracts. The oak leaves extract presents the highest antioxidant capacity followed by the eucalyptus extract. The results obtained by these two methods are comparable and show a similar trend in most of the cases. Yet the results obtained for white thyme diverged between the F–C and DPPH methods. These differences can be related to the different reagents, mechanisms, and reaction media used in both assays; it is thus possible that complex natural matrices such as plant extracts behave different in each assay [31].

The group of extracts with low reducing power ranges from common thyme, with medium reducing power, to *S. muticum*, with a very low reducing power. In this case, data from F–C and DPPH assays also show some divergences.

The obtained results present a good general agreement, demonstrating that both methods are suitable to characterize the antioxidant capacity of the plant extracts (see Figure S1 in the supplementary information).

#### 3.1.2. Antioxidant Capacity Determination by Synthesis of Silver Nanoparticles

Özyürek and co-workers [35] developed a method for the determination of the antioxidant capacity of natural samples based on the synthesis of silver NPs. Metal nanoparticles dispersed in liquid media present an UV-Vis absorption band, which is known as surface plasmon resonance (SPR) band [8,39]. The method proposed by Özyürek et al. establishes that growth of silver nanoparticles, but not nucleation, is responsible of the absorption increase observed for their SPR band. Therefore, in the proposed method, monodisperse silver seeds particles are synthesized by reduction of AgNO_3_ using a weak reductant (sodium citrate). The absorbance of this initial silver seeds solution (*A_0_*) is determined at 423 nm and proved to be constant with time. Then, the antioxidant capacity of the extracts is measured by mixing the silver seeds with each extract, and determining the absorbance of the solution. The color evolution of each mixture shows the growth of the silver nanoparticles and the increment of absorbances (*∆A*) is then related to the capacity of each extract to reduce metals.

A comparison among the three antioxidant test can be found in the supplementary information (Figure S1).

(a) Kinetics of nanoparticles growth

**Figure 2:**
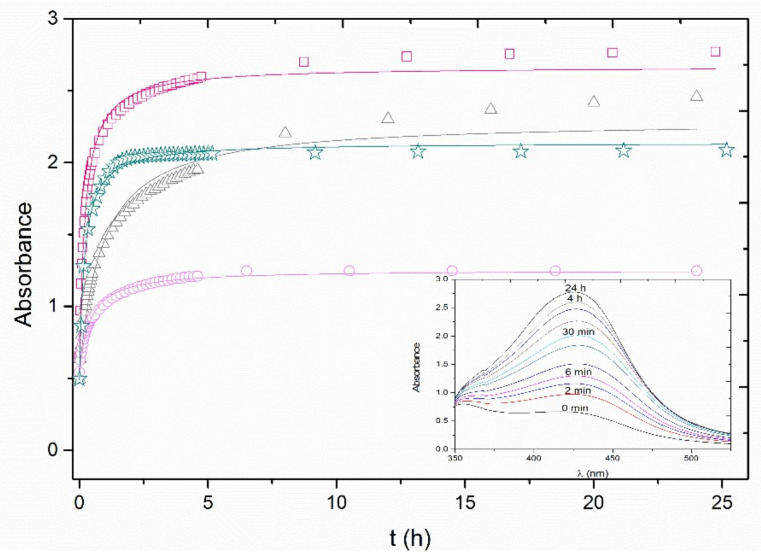
Absorption kinetics of the silver NPs’ growth with four different extracts: white thyme (squares), eucalyptus (triangles), oak (stars), and white cedar (circles). Inset shows the evolution of the white thyme absorption spectrum with time. Data were fitted to a second order model.

The method proposed by Özyürek and co-workers [35] that was developed with commercial antioxidant reagents (e.g., gallic acid, ascorbic acid), establishes a reaction time of 30 min. As different antioxidant compounds are used in this work, a preliminary kinetic study was carried out (*V_extract_* = 0.05 mL; 1.8% total volume). Three of the extracts that presented the highest antioxidant capacity (oak, eucalyptus, and white thyme) were selected for these kinetic experiments, together with white cedar, as an example of a low reducing power extract. The maximum height of the SPR band evolution over time of the selected extracts was investigated (Figure 2).

As expected, the maximum of the SPR band continues to growth after 30 min (inset of Figure 2). Among the four extracts selected, the fastest evolution of the SPR was observed with the oak extract, which reached equilibrium in about 2 h. White cedar required ca. 4 h, whilst the eucalyptus and white thyme extracts required longer times to achieve an equilibrium of 8 and 15 h, respectively. The evolution of absorption spectra over time for silver NPs growth using oak, eucalyptus, and white cedar extracts is shown in the supplementary material (Figure S2). A reaction time of 24 h was chosen to perform silver nanoparticle growth experiments, ensuring that the equilibrium is attained in all cases.

The data obtained were fitted to a second order kinetic model (Equation (1)).

(1) $$\frac{dC}{dt}=-kC^{2}$$

where *C* is the concentration of silver nanoparticles in mol·L^−1^, *k* is the rate constant in L·mol^−1^·h^−1^, and *t* is time in h.

Considering that the relationship between the absorbance and the concentration can be expressed as:

(2) $$\frac{C}{C_{0}}=\frac{A-A_{\infty }}{A_{0}-A_{\infty }}$$

where *C*_0_ is the initial concentration of silver seeds in mol·L^−1^ and *A*, *A_0_*, and *A_∞_* are the absorbance at time *t*, the initial absorbance, and the absorbance at infinite time, respectively.

The integration of Equation (1), taking into account Equation (2), results in the following expression:

(3) $$A=\frac{A_{0}+k^{\prime }A_{\infty }t}{1+k^{\prime }t}$$

where *k′* is a kinetic constant expressed in h^−1^, which comprises the rate constant of the NPs growth (*k*) and the initial concentration of silver seeds in solution (*C_0_*).

Equation (3) fitted well with the experimental data for oak and white cedar extracts, but not for white thyme and eucalyptus data, especially at long time values. This fact is related to the simplicity of the model, which does not consider, e.g., the role of organic matter in the growth of silver nanoparticles. Table 1 shows the values of the constant k′ for the growth of Ag NPs with the four extracts tested. In agreement with the observations, oak shows the fastest reaction time while eucalyptus is the slowest.

(b) Influence of the extract concentration on the nanoparticle growth

**Figure 3:**
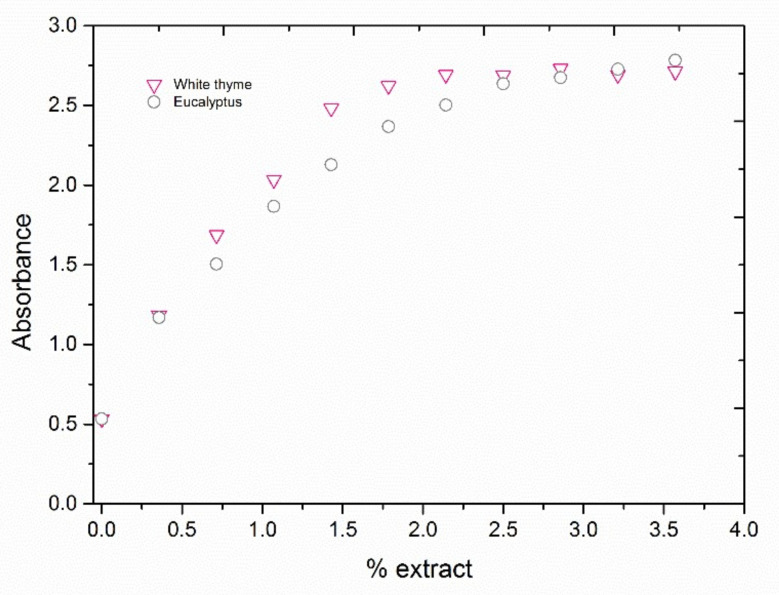
Influence of extract percentage in silver NPs’ growth.

Once the reaction time was determined, several experiments were done to determine the optimal amount of extract that provides the maximum absorbance values, i.e., to produce the maximum nanoparticles growth. White thyme and eucalyptus extracts were chosen due to their high absorbance values observed during the kinetic studies. Experiments were carried out by varying the percentage of extract added to a silver seed mixture and determining the absorbance at the maximum height of the SPR band, as shown for eucalyptus and white thyme extracts in Figure 3. A linear increase of absorbance was observed until extract percentages approached 1.5% (*v*/*v*). Then, the growth was progressively decreased until the extract percentages approached a value of 2. At that percentage, a stabilization of the absorbance was observed, especially for the mixture of silver NPs reduced by the white thyme extract. The SPR band maximum absorbance values do not vary significantly for extracts percentages above 1.8%; thus, this amount was selected to carry out the experiment of silver nanoparticles growth.

(c) Antioxidant capacity studies and characterization of nanoparticles

**Figure 4:**
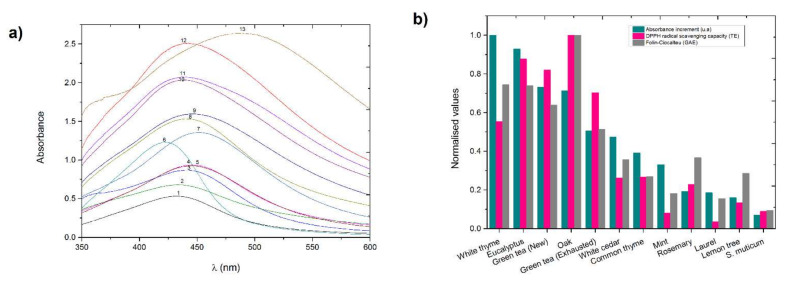
Silver NPs’ growth with plant extracts (0.05 mL). (**a**) Absorption spectra: 1—silver seeds (SNP); 2—*S. muticum*; 3—lemon; 4—laurel; 5—rosemary; 6—mint; 7—common thyme; 8—white cedar; 9—green tea (exhausted) (GTE); 10—Oak; 11—green tea (new) (GTN); 12—eucalyptus; 13—white thyme. (**b**) Comparison of absorbance increments of NPs’ growth with Folin–Ciocalteu and DPPH data. Normalized values respect to the maximum value of each experiment.

The SNPAC experiments were done at the optimal reaction time and extract percentage using the 11 plant extracts. Figure 4a shows the absorption spectra of the growth of initial silver seeds with the different natural compounds studied.

It is worth noting that a shift in absorption maxima, with respect to the maximum of initial silver seeds, was observed for some of the extracts. Namely, a red shift was observed for the mint, and a blue shift was observed for white thyme. Moreover, another peak around 360 nm was observed in the white thyme spectrum. The rest of materials had the absorption maximum in a similar position than silver seeds; a slight blue shift was observed for common thyme, white cedar, and green tea (exhausted) extracts, but it was not as important as the shift observed for the white thyme extract. This behavior has already been noticed in the literature and indicates size and shape transformations of the formed silver NPs [40,41,42].

The data in Figure 4a were used to calculate the absorbance increments (*∆A*) produced by each extract. The silver seeds absorbance (0.533 a.u.) was taken as *A_0_*. Figure 4b represents normalized ∆*A*, F–C, and DPPH data. Normalized ∆*A* data were calculated with respect to the maximum value, which was observed in white thyme (2.1 a.u.). In terms of silver nanoparticle growth and concurring with F–C and DPPH results, two groups of extracts can be distinguished. The first group represents extracts with high capacity to make the nanoparticles grow. This group consists of white thyme, eucalyptus, green tea (new), oak, and green tea (exhausted). The other extracts, which show a lower capacity to make the silver nanoparticles grow, are included in the second group. Figure 4b allows the comparison of the three methods described to estimate the antioxidant capacities of the extracts. A general trend is observed where those extracts with high antioxidant capacity, determined by DPPH and F–C methods, provided large increments of absorbance when measured by the SNPAC method. Those extracts with enhanced antioxidant capacity are the white thyme, eucalyptus, oak, and both tea extracts. All the other extracts show moderate to low DPPH, F–C, and also SNPAC values.

**Figure 5:**
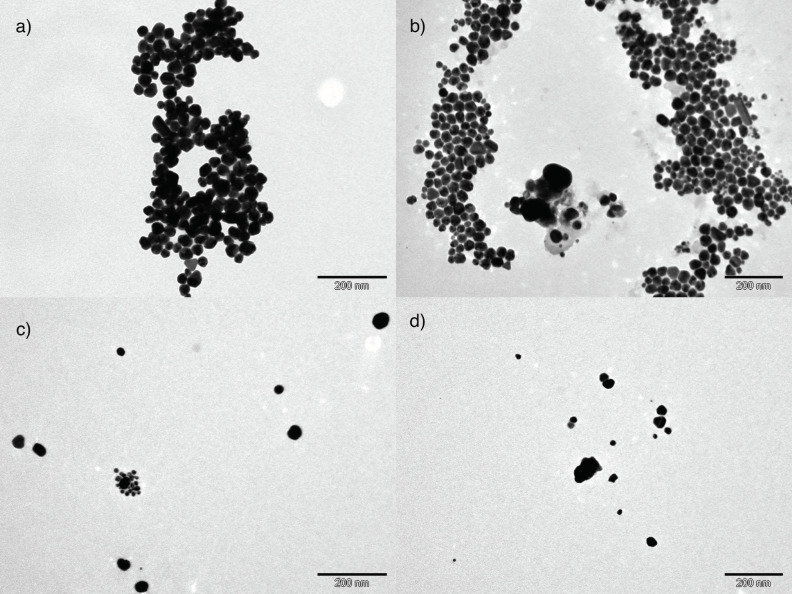
TEM images of silver NPs synthesized with extracts by SNPAC method: (**a**) white thyme; (**b**) eucalyptus; (**c**) oak; (**d**) white cedar.

These results agree with TEM micrographs of the synthesized silver nanoparticles (Figure 5).

Images of silver NPs obtained with white thyme and eucalyptus extracts (Figure 5a,b) show large amounts of nanoparticles forming groups. Hexagonal shapes are predominant, but spherical, triangular, and rod forms were also observed. TEM images show sizes around 50 nm. Images for synthesized NPs with oak extract (Figure 5c) also show significant amounts of nanoparticles, but they appeared more dispersed throughout the sample. The size of silver NPs obtained with oak extract is also somehow smaller than 50 nm. This observation could be an indication of a more important capping effect of the oak extract maintaining silver NPs smaller, and at the same time, dispersed in solution. Finally, in the images of nanoparticles obtained with a moderate reductant, white cedar (Figure 5d), a smaller amount of NPs were observed, most of them with sizes below 50 nm. In the supplementary material, the panoramic views of the silver NPs obtained with these four extracts are shown (Figure S3) together with the histograms corresponding to the particle size for each solution (Figures S4 and S5).

**Figure 6:**
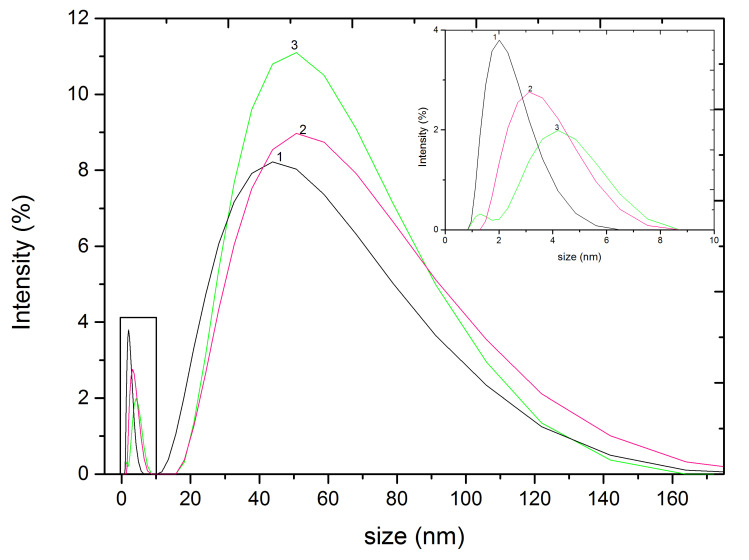
Dynamic Light Scattering particle size distribution (by intensity) of silver NPs synthesized with extracts: 1—Silver seeds (SNP); 2—White cedar; 3—White thyme. Inset shows the peak distribution corresponding to the NPs with sizes below 8 nm.

The size distribution of the silver NPs formed was obtained by DLS measurements (Figure 6).

Two particle size distributions were observed, one around 2 nm and the other around 57 nm (Figure 6). Particles with sizes around 57 nm were also observed in TEM micrographs (Figure 5). However, the smaller particles (2 nm) were not noticed. This fact could be related to the resolution of the TEM equipment. The smaller NPs might not be observed by the instrument or they could be covered by the larger silver NPs. If we compare the initial silver seeds with the silver NPs synthesized with a strong reductant extract (white thyme) and a moderate reductant extract (white cedar), we observe an intensity increase in the peak corresponding to the larger nanoparticles, especially remarkable in the case of white thyme. Accordingly, a decrease was observed for the peak of the smaller nanoparticles fraction, which was shifted towards larger particles (centered at ca. 4 nm). This observation is in agreement with the changes observed in the absorption spectrum of silver seeds after its reduction by natural extracts (Figure 4). The DLS diagrams for silver NPs growth with eucalyptus and oak extracts are shown in the supplementary material (Figure S6).

### 3.2. Synthesis and Characterization of Metallic Nanoparticles

The reducing power of the eucalyptus extract was tested for the synthesis of metallic nanoparticles. This extract presents one of the highest antioxidant values obtained by both traditional and SNPAC methods. In addition, eucalyptus is a foreign and very spread species in Galician forests, thus the use of the leaves constitutes a waste recovery. Five metals, copper, iron, lead, nickel, and silver were selected for the experiments. The addition of each metal salt to the extract produced mixtures with different colors. An image of the mixtures metal salts–eucalyptus extract is shown in the supplementary material (Figure S7).

**Figure 7:**
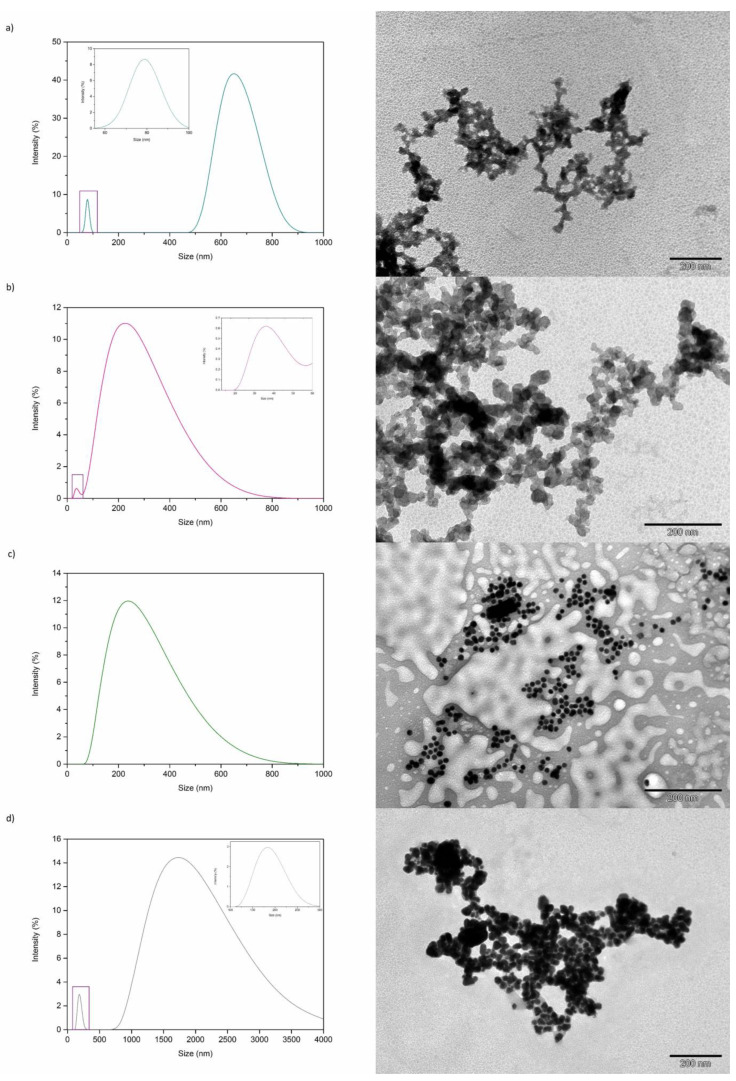
Synthesis of nanoparticles with eucalyptus extract and Cu(NO_3_)_2_·3H_2_O, Fe(NO_3_)_3_·9H_2_O, Ni(NO_3_)_2_·6H_2_O, and AgNO_3_ 0.1 M at 25 °C: TEM images and DLS diagram. (**a**) Copper NPs; (**b**) iron NPs, (**c**) nickel NPs, and (**d**) silver NPs.

The synthesis was carried out by mixing the metal salts with the same volume of eucalyptus extract [12,17]. Then, the obtained nanoparticles were characterized by TEM and DLS. Figure 7 shows TEM images and DLS size distributions for copper, iron, nickel, and silver NPs synthesized using the eucalyptus extract. Regarding TEM characterization, results for all the metals tested show the formation of a large amount of NPs with heterogeneous shapes and sizes around 50 nm. However, the separation between the NPs is not as good as the one obtained with the NPs produced using the SNPAC method, except for the Ni ones (Figure 4). The lack of separation is in agreement with the results observed in the DLS diagrams, where the main size distribution for all metals is above 100 nm. Peaks with sizes below 100 nm are also observed for Cu, Fe, and Ag NPs. These peaks, which match with particles size observed in TEM images, appear at a much lower frequency than the one corresponding to the larger size distribution. This is a common problem in DLS measurements, where agglomerated clusters of NPs can be wrongly interpreted to be single individual NPs [43]. As mentioned above, TEM micrographs for nickel NPs show the formation of a large number of particles with a small size (<50 nm) that are much more separated than the NPs of the other metals tested. This fact indicates that eucalyptus extract is not only a good reductant, but also a suitable capping agent for obtaining nickel NPs.

Regarding Pb, the mixture with the extract leads to the formation of a precipitate. TEM images for this mixture show the appearance of aggregates in solution, but NPs are not observed. Besides, DLS size distributions for lead samples do not show reproducible results; this could be related to the fact that there are no NPs in solution, and only Pb precipitates are formed. TEM and DLS data for lead mixtures are shown in the supplementary material (Figure S8). These results show that even though the eucalyptus extract is capable of forming Cu, Fe, Ni (histogram in Figure S9), and Ag NPs, it is not a suitable reductant to synthesize Pb NPs. This fact is in agreement with the existing literature, where it is shown that plant extracts may not produce NPs with every metal salt [20].

## 4. Conclusions

Hot water extraction using different bio-based products constitutes a quick, green, and cost-effective process to obtain extracts with potential high antioxidant/reducing capacity. The antioxidant capacity of these extracts can be easy characterized using different methods available. In our study, we have tested three of these methods: the Folin–Ciocalteu, the DPPH radical scavenging capacity, and the SNPAC method. The former two are both traditional tests to estimate the antioxidant capacity of different substances, while the third one estimates the antioxidant capacity based in silver nanoparticle formation. The results obtained using these three methods showed similar trends when plant extracts are analyzed using either one of them. This demonstrates that any of these three assays can be used to quickly test extracts and identify those that might constitute an adequate reactant to develop the synthesis of nanoparticles avoiding the use of hazardous chemicals employed in other synthesis of nanoparticles.

The kinetics of the nanoparticle growth as well as the effect of the antioxidant concentration were evaluated in order to adjust the SNPAC method to the bio-based reductants (plant extracts). The characterization of the obtained silver suspensions by TEM and DLS showed the formation of silver nanoparticles of different sizes and shapes and allowed demonstrating the potential of the chosen plant extracts as alternative reagents for nanoparticle synthesis.

An assay for synthesis and characterization of metal nanoparticles was developed using a eucalyptus extract which was among the ones that showed the highest results in the three antioxidant tests employed. Copper, iron, nickel, and silver NPs were successfully obtained by mixing salt solutions of these four metals with a water extract of eucalyptus leaves. However, the extract was not appropriate to synthesize Pb NPs. As it could be expected, not only the properties of the extracts must be considered in nanoparticle formation and the nature of the metal cation employed must also be taken into consideration. Further optimization of nanoparticle formation should be studied in the future, since most of the NPs obtained appeared in solution as aggregates, especially iron and copper ones. On the contrary, silver and nickel showed very promising, straightforward results for nanoparticle formation with a simple mixing process.

## Figures and Tables

**Table 1:** Kinetic constants of silver NPs’ growth.

Material	*k′* (h^−1^)
White thyme	3.6 ± 0.2
Eucalyptus	0.7 ± 0.1
Oak	5.5 ± 0.3
White cedar	1.9 ± 0.1

## Data Availability

The datasets generated during the study are available from the corresponding authors on request.

## Supplementary Materials

The following are available online at https://www.mdpi.com/article/10.3390/nano11071679/s1, Figure S1. Comparison of the water extract values obtained with the three antioxidant methods studied, Figure S2: Kinetic study of the spectrum of silver NPs growth with oak (a), eucalyptus (b) and white cedar extracts (c). Mixture: 2 mL of silver seeds, 0.75 mL of H_2_O and 0.05 mL of extract, Figure S3: TEM images of silver NPs synthesized with extracts in SNPAC method: (a) white thyme; (b) eucalyptus; (c) oak; (d) white cedar. Panoramic views, Figure S4. Histogram of particle size corresponding to pictures shown in Figure 5 in main manuscript, Figure S5. Histogram of particle size corresponding to pictures shown in Figure S3 of the Supplementary information, Figure S6: Dynamic Light Scattering diagram. Size distribution by intensity of silver NPs synthesized with extracts: 1—Silver seeds (SNP); 2—Eucalyptus; 3—Oak. Inset shows the peak distribution corresponding to the NPs with sizes below 10 nm, Figure S7: Mixtures of eucalyptus extract with different metal salts, Figure S8: Synthesis of nanoparticles with eucalyptus extract and Pb(NO_3_)_2_ 0.1 M at 25 °C: TEM images and DLS diagram, Figure S9. Histogram of Ni particle size corresponding to picture shown in Figure 7c of the main manuscript.
