# Supplementary material for: Antioxidant Capacity Assessment of Plant Extracts for Green Synthesis of Nanoparticles
Source: Nanomaterials (Basel). 2021 Jun 25;11(7):1679. doi: 10.3390/nano11071679 (PMC8308177; doi:10.3390/nano11071679)
Supplement: Supplementary file 1 [file nanomaterials-11-01679-s001.zip › nanomaterials-1205094-supplementary.pdf]

SUPPLEMENTARY MATERIAL

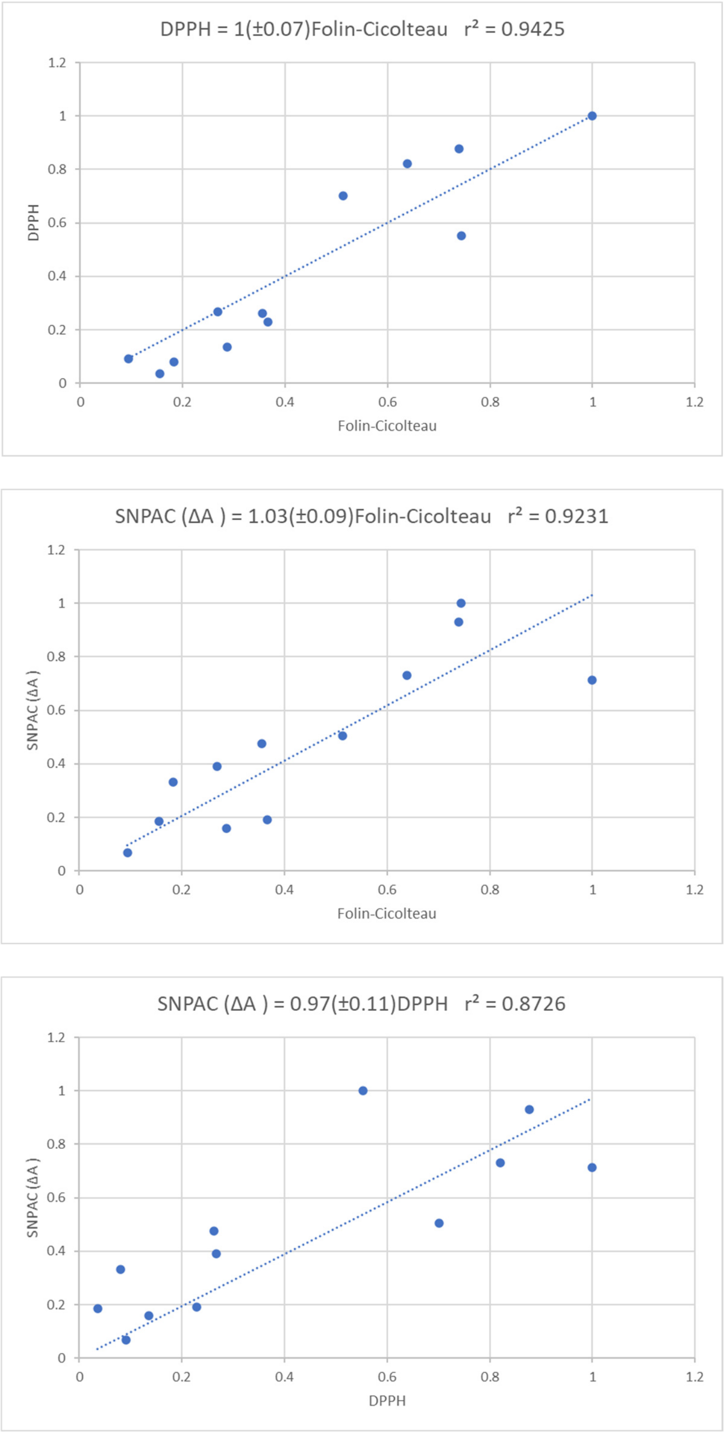

Figure S1. Comparison of the water extract values obtained with the three antioxidant methods studied. Methods were compared with each other. Fitting line was forced through the origin. In parenthesis, the error in the slope obtained for each fitting.

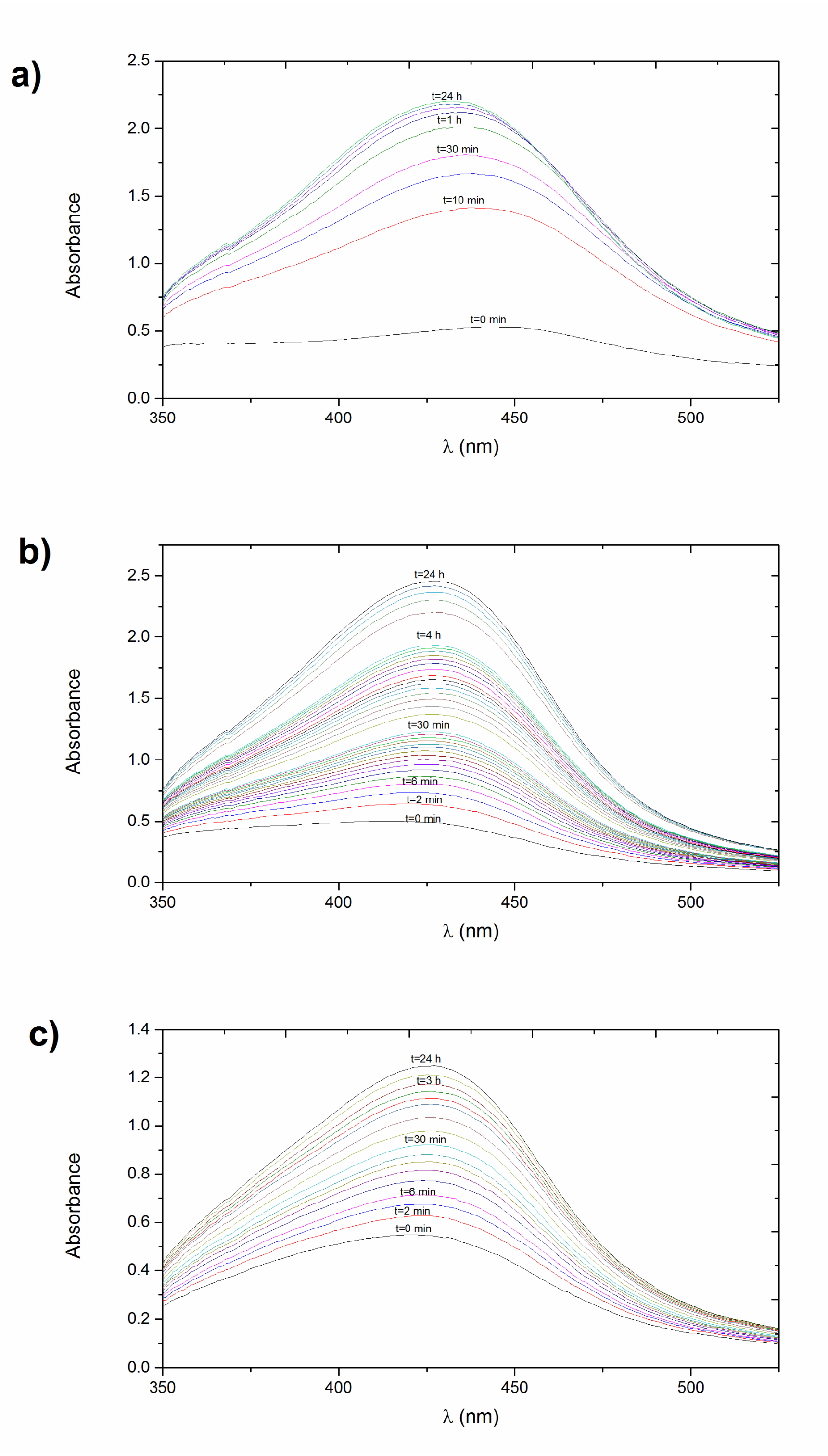

Figure S2. Kinetic study of the spectrum of silver NPs growth with oak (a), eucalyptus (b) and white cedar extracts (c). Mixture: 2 mL of silver seeds, 0.75 mL of H_2O and 0.05 mL of extract.

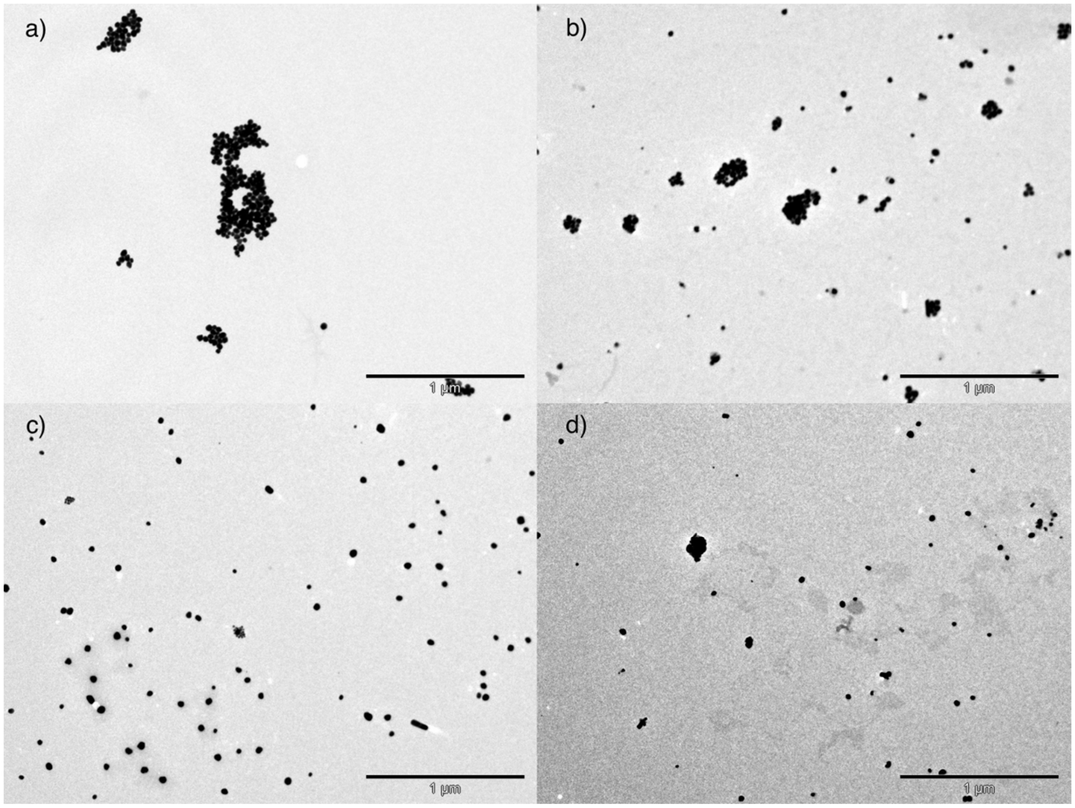

Figure S3. TEM images of silver NPs synthesized with extracts in SNPAC method: a) white thyme; b) eucalyptus; c) oak; d) white cedar. Panoramic views.

As it was mentioned in the text (section 3.1.2 section c) both white thyme and eucalyptus produce a large amount of silver NPs and they use to appear grouped in solution. Oak extract also produces an important amount of NPs, however they are more separated in solution. This effect is more noticeable in TEM panoramic views. As regard white cedar, the amount of synthesized NPs is lower than the other three extracts.

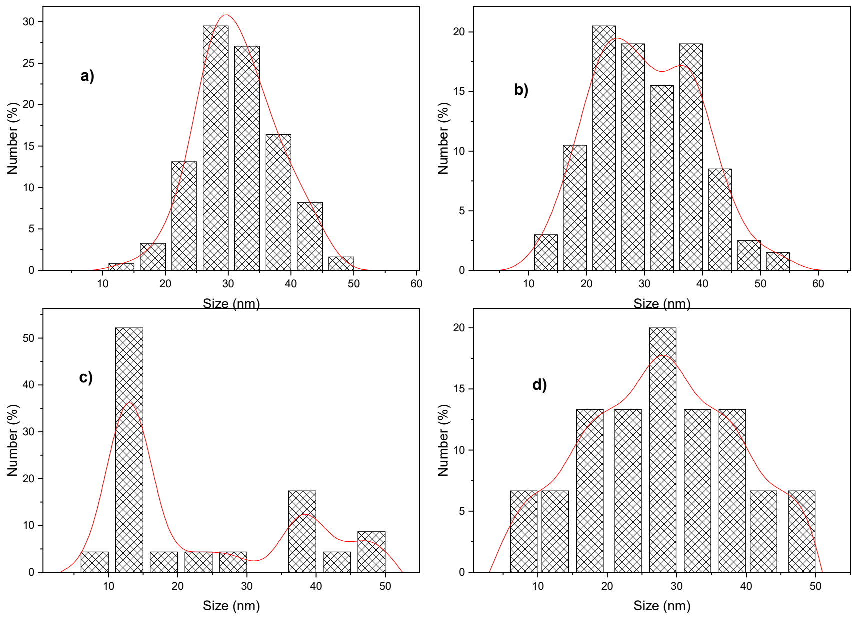

Figure S4. Histogram of particle size corresponding to pictures shown in Figure 5 in main manuscript.

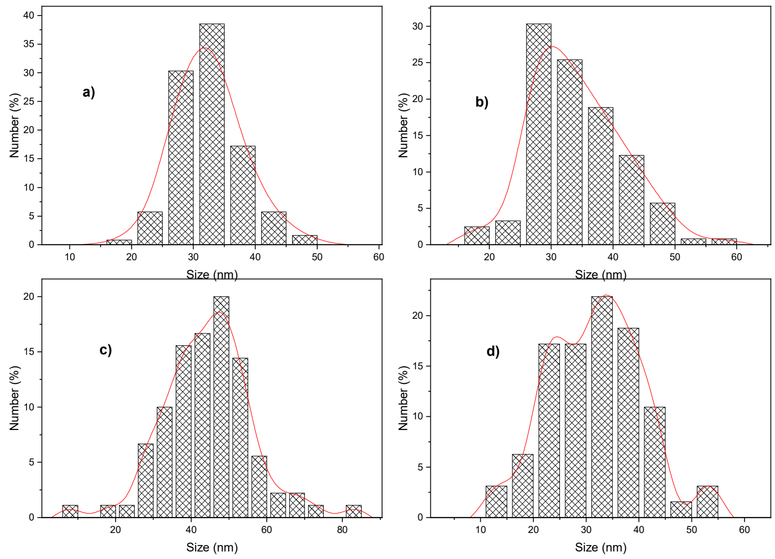

Figure S5. Histogram of particle size corresponding to pictures shown in Figure S3 of the Supplementary information.

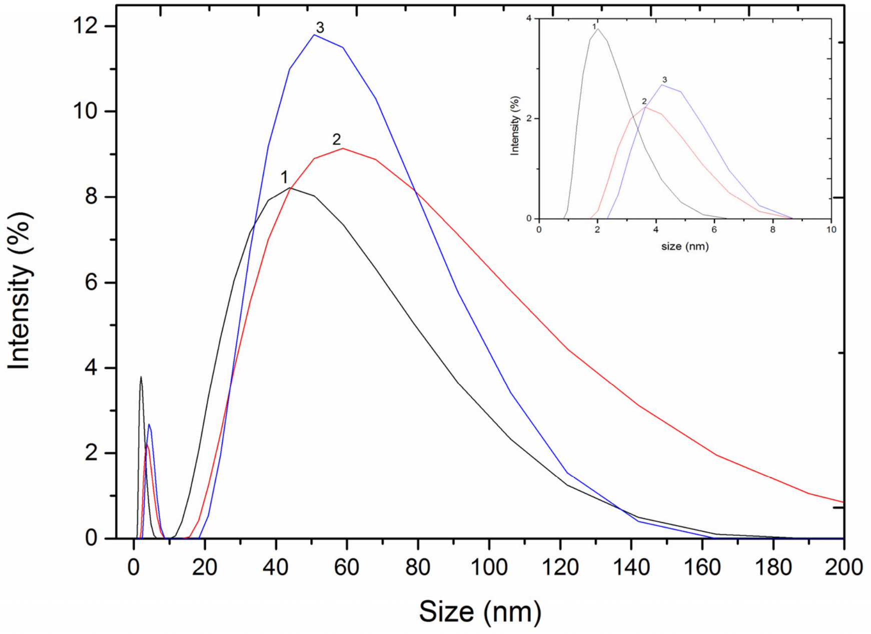

Figure S6. Dynamic Light Scattering diagram. Size distribution by intensity of silver NPs synthesized with extracts: 1- Silver seeds (SNP); 2- Eucalyptus; 3- Oak. Inset shows the peak distribution corresponding to the NPs with sizes below 10 nm.

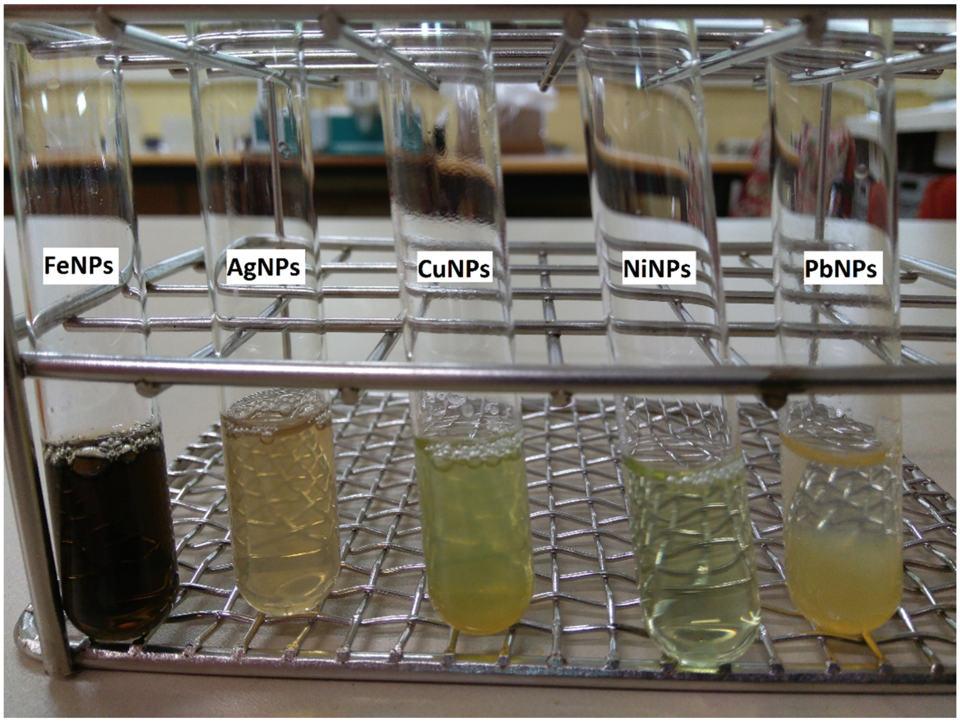

Figure S7. Mixtures of eucalyptus extract with different metal salts.

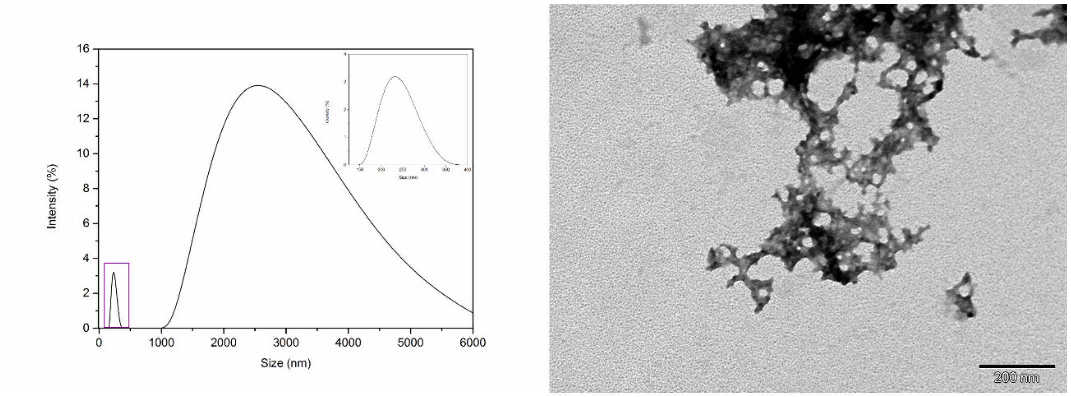

Figure S8. Synthesis of nanoparticles with eucalyptus extract and $Pb(NO_3)_2$ 0.1 M at 25 °C: TEM images and DLS diagram.

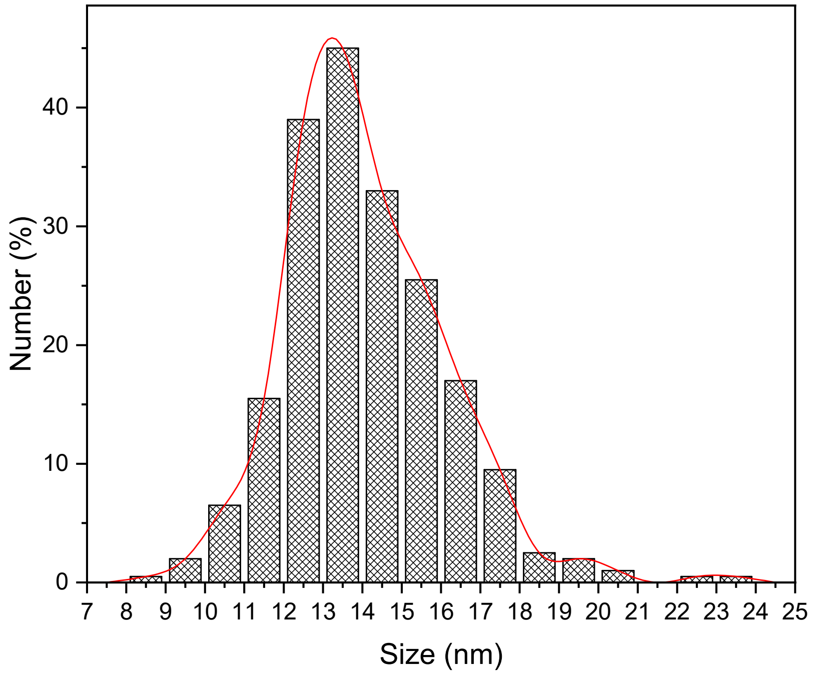

Figure S9. Histogram of Ni particle size corresponding to picture shown in Figure 7c of the main manuscript.
